# Cytotoxicity of Isoxazole Curcumin Analogs on Chronic Myeloid Leukemia-Derived K562 Cell Lines Sensitive and Resistant to Imatinib

**DOI:** 10.3390/ijms24032356

**Published:** 2023-01-25

**Authors:** Giordana Feriotto, Paolo Marchetti, Riccardo Rondanin, Federico Tagliati, Serena Aguzzi, Simone Beninati, Fabio Casciano, Claudio Tabolacci, Carlo Mischiati

**Affiliations:** 1 Department of Chemical, Pharmaceutical and Agricultural Sciences, University of Ferrara, 44121 Ferrara, Italy; 2 Department of Neuroscience and Rehabilitation, University of Ferrara, 44121 Ferrara, Italy; 3 Department of Biology, University of Rome “Tor Vergata”, 00133 Rome, Italy; 4 Department of Translational Medicine and LTTA Centre, University of Ferrara, 44121 Ferrara, Italy; 5 Department of Oncology and Molecular Medicine, Istituto Superiore di Sanità, 00161 Rome, Italy

**Keywords:** imatinib, CML, curcumin, derivatives, apoptosis

## Abstract

Despite curcumin (CUR) inhibiting cell proliferation in vitro by activating apoptotic cell death, its use in pharmacological therapy is hampered by poor solubility, low stability in biological fluids, and rapid removal from the body. Therefore, CUR-derivatives with better biological and chemical–physical characteristics are needed. The bis-ketone moiety of CUR strongly influences its stability in slightly alkaline solutions such as plasma. Here, we considered its replacement with isoxazole, beta-enamine, or oxime groups to obtain more stable derivatives. The evaluation of the chemical–physical characteristics showed that only of the isoxazole derivatives **2** and **22** had better potential than CUR in terms of bioavailability. The UV–visible spectrum analysis showed that derivatives **2** and **22** had better stability than CUR in solutions mimicking the biological fluids. When tested on a panel of cell lines, derivatives **2** and **22** had marked cytotoxicity (IC50 = 0.5 µM) compared with CUR only (IC50 = 17 µM) in the chronic myeloid leukemia (CML)-derived K562 cell line. The derivative **22** was the more selective for CML cells. When administered at the average concentration found for CUR in the blood of patients, derivatives **2** and **22** had potent effects on cell cycle progression and apoptosis initiation, while CUR was ineffective. The apoptotic effect of derivatives **2** and **22** was associated with low necrosis. In addition, derivative **22** was able to reverse drug resistance in K562 cells resistant to imatinib (IM), the reference drug used in CML therapy. The cytotoxicity of derivative **22** on IM-sensitive and resistant cells was associated with upregulation of *FOXN3* and *CDKN1A* expression, G2/M arrest, and triggering of apoptosis. In conclusion, derivative **22** has chemical–physical characteristics and biological effects superior to CUR, which allow us to hypothesize its future use in the therapy of CML and CML forms resistant to IM, either alone or in combination with this drug.

## 1. Introduction

Chronic Myeloid Leukemia (CML) is a myeloproliferative disease caused by a translocation between chromosomes 9 and 22 (called Philadelphia chromosome) resulting in the fusion of two genes, ABL1 (Ch9) and BCR (Ch22). The chimeric product of this fusion (BCR-ABL oncogene) is a protein with constitutive tyrosine kinase activity leading to uncontrolled proliferation, protection from apoptosis, and promotion of invasion. Although the development of BCR-ABL1 tyrosine kinase inhibitors (TKIs) such as imatinib (IM), dasatinib, and nilotinib has led to significant progress in CML therapy, resistance occurs in patients with advanced disease [1]. IM is a potent and selective inhibitor of the Bcr-Abl protein tyrosine kinase approved for the treatment of CML. In pharmacokinetic studies in healthy volunteers and CML patients, IM reached plasma concentrations between 0.4 and 2 µM. However, these values were not always sufficient to exceed the in vitro tyrosine kinase inhibitory concentration (1 µM) and to normalize the hematologic parameters in the patients [2].

Curcumin (CUR), a natural compound present in turmeric (*Curcuma longa* L.), is a promising therapeutic agent currently being tested in several human clinical trials [3]. Many studies have shown its anti-proliferative and anti-carcinogenic activities on animal and human cell lines [4,5]. Although it was very promising in terms of anti-cancer potential [6], the molecule had limited intestinal absorption, was poorly soluble in aqueous solutions [7], and unstable due to the breaking of the bonds between C4 and C5 [8]. Indeed, in biological fluids, the molecule undergoes structural modifications, such as the shift of the keto-enol tautomer towards the enol form [9,10], and the conversion into ferulic acid and feruloyl methane and then into vanillin and acetone [8,11], with renal elimination of by-products [12]. Furthermore, CUR undergoes glucuronidation on the hydroxyls present in the aromatic ring by UDP-glucuronosyl transferase, whereby it is excreted from the body [13,14]. Due to these limitations, the blood levels observed in human clinical studies ranged from 0.4 to 3.6 μM [15]. Since CUR showed antiproliferative activity in the concentration range between 5-80 μM in in vitro assays [3], the hematic level of CUR did not appear sufficient to obtain a therapeutic result.

Therefore, CUR-derived analogues and delivery systems have been proposed in an attempt to improve its bioavailability [16,17,18,19]. One of the aspects to take into consideration in improving the bioavailability of CUR is its stability in biological weakly basic pH solutions. The instability of the molecule was due to the bis-ketone moiety [9], wherein C4 becomes deprotonated at slightly alkaline pH, causing the bond between C4 and C5 to break and the by-products to form [8,20,21]. Therefore, the replacement of the bis-ketone moiety with groups capable of decreasing the acidity of the hydrogen in C4 could be useful to confer ameliorated stability to the CUR-derivatives. 

In this study, to obtain more stable derivatives, bis-ketone substitutions with isoxazole ring, β-enamine ketones and oximes in the CUR molecule were considered. The stability of the CUR-derivatives in biological fluids and the drug-like characteristics were studied. Cytotoxicity and apoptosis-triggering effects of the derivatives were also studied in CML-derived K562 cell lines sensitive or resistant to IM.

## 2. Results

### 2.1. The Stability of CUR and Its Derivatives

**Figure 1:**
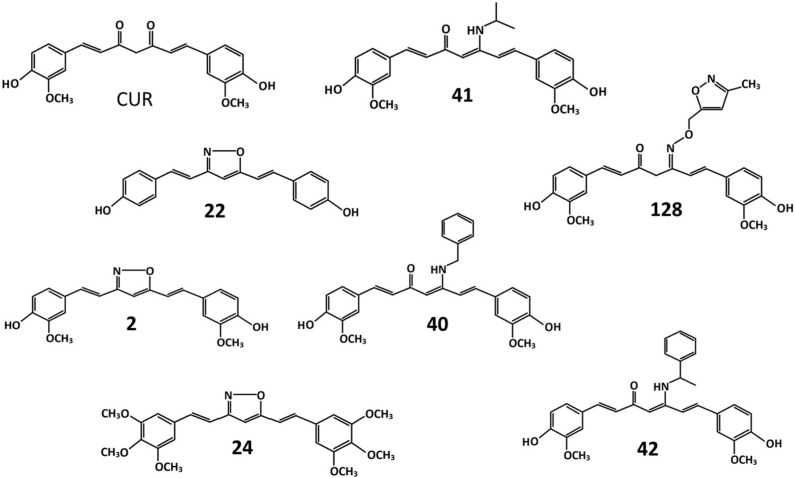
Chemical structures of curcumin and its derivatives. **CUR**: 1,7-bis(4-hydroxy-3-methoxyphenyl)-1,6-heptadiene-3,5-dione. **2**: 4-(2-{5-[2[2-(4-hydroxy-3-methoxyphenyl)ethenyl]-1,2-oxazol-3-yl}ethenyl)-2-methoxyphenol. **22**: 4-(2-{5-[2-(4-hydroxyphenyl)ethenyl]-1,2-oxazol-3-yl}ethenyl)phenol. **24**: 3,5-bis[2 -(3,4,5-trimethoxyphenyl)ethenyl]-1,2-oxazole. **40**: 5-(benzylamino)-1,7-bis(4-hydroxy-3-methoxyphenyl) hepta-1,4,6-trien-3-one. **41**: 1,7-bis(4-hydroxy-3-methoxyphenyl)-5-[(propan-2-yl)amino]hepta-1,4,6-trien-3-one. **42**: 1,7-bis(4-hydroxy-3-methoxyphenyl)-5-[(1-phenylethenyl)amino]hepta-1,4,6-trien-3-one. **128**: 1,7-bis(4-hydroxy-3-methoxyphenyl)-5-{[(3-methyl-1,2-oxazol-5-yl)methoxy]imino}hepta-1,6-dien-3-one.

The replacement of the bis-ketone moiety with groups capable of reducing the acidity of the C4-H present in CUR could minimize the risk of breaking the bond between C4 and C5, thus conferring stability to the molecule. Moreover, in our previous studies, the transformation of the bis-ketone of CUR to an isoxazole ring led to derivatives with improved stability toward nucleophilic reactions [22]. Therefore, the molecules shown in Figure 1 were studied.

The isoxazole, β-enamine ketone and oxime derivatives were produced using a synthetic procedure similar to that described in [23]. In derivatives **2**, **22**, and **24**, the bis-ketone was replaced by an isoxazole. In derivative **24**, the introduction of three methoxy groups at the 3, 4, and 5 position of each benzene ring resulted in the removal of the 4-hydroxyl group. This modification should preserve the derivative from glucuronidation, lengthening its permanence in the bloodstream [14]. In derivatives **40**, **41**, and **42**, the bis-ketone has been replaced by a β-enamine ketone, and in derivative **128,** by an oxime. All molecules were dissolved in dimethyl sulfoxide (DMSO).

**Figure 2:**
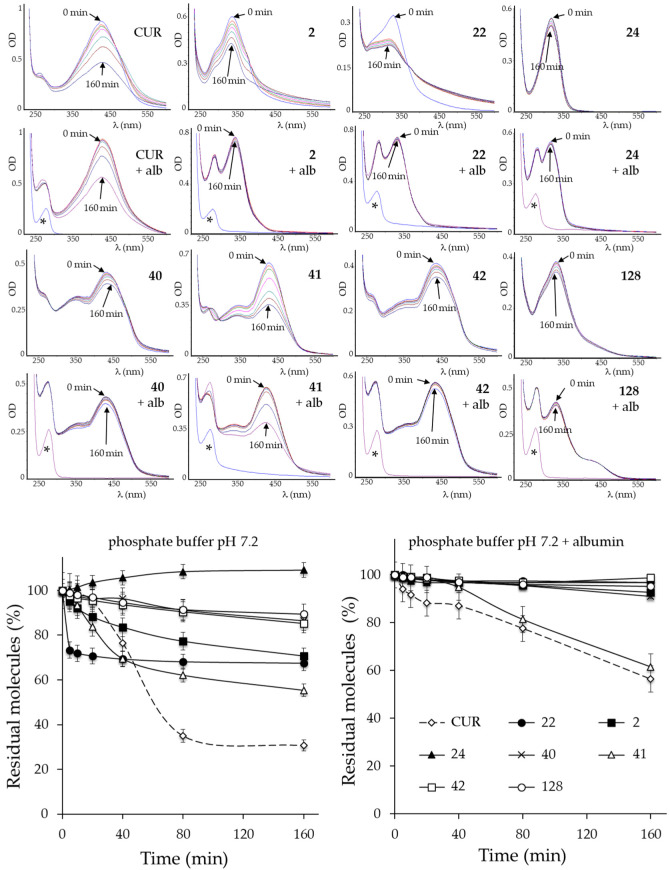
UV–visible spectrum analysis of CUR and its derivatives. (**Upper side)**: Time-dependent modification of the molecule in simple phosphate buffer pH 7.2 solution with or without albumin (alb). Representative spectra are shown. Asterisks indicate the spectrum profile of a blank solution (phosphate-buffered solution of albumin). (**Lower side**): Graphical representation of the residual molecules present at each time point, estimated as the ratio between the absorbance value of the maximum absorption peak at the indicated time and the value at time zero, and expressed as a percentage. The indicated values are the arithmetic means ± SD (n = 4).

The stabilities of CUR and its derivatives were studied in aqueous solutions at physiological pH, in the presence or absence of albumin. For this aim, the modifications of the UV–visible absorption spectrum at different incubation times were evaluated. The optical density values relating to the peak of greatest absorption of each derivative were studied to compare the stability of the derivatives. The values in each time point were expressed as a percentage with respect to the value observed at the initial time (Figure 2).

The correlation between the stability of the molecule and its structure has given better knowledge of which molecular substitutions confer greater stability to the derivatives. This information could be useful for the design of stable second-generation molecules with increased cytotoxicity.

Derivatives **24**, **40**, **42**, **128** were stable molecules even in the absence of albumin, while almost all other derivatives, with the exception of **41**, were more stable than CUR in the presence of albumin. This feature suggested their possible stability in biological fluids such as plasma. Substitution of the bis-ketone moiety with isoxazole rings, β-enamine ketones or oximes were present in these molecules. Therefore, all substitutions increased the stability of the derivative and could serve for the future design of stable second-generation molecules with improved cytotoxicity. In addition, the simultaneous modification of groups linked to the aromatic rings or the opening of the isoxazole ring reduced their stability. For example, among the derivatives with β-enamine ketone (**40**, **41** and **42**), only the derivative with a small functional group bonded to N (**41**, isopropyl) was unstable, while the others with a bulkier group (**40**, toluene; **42**, ethylbenzene) were stable even in the absence of albumin. Consequently, the introduction of a large N-linked group appears to be a prerequisite for obtaining future stable β-enamine ketones.

### 2.2. The Cytotoxicity of CUR and Its Derivatives

In previous work, we tested the cytotoxicity of CUR and derivatives **2**, **40**, **41**, and **42** in HA22T/VGH primary liver cancer and multidrug-sensitive (MCF7) and resistant (MCF7R) breast cancer cell lines [23]. In this work, we additionally tested the cytotoxicity of CUR and its derivatives indicated above on a larger panel of cancer cell lines, including K562 (CML), TE85 (osteosarcoma), MG63 (osteosarcoma), TT (thyroid carcinoma), Colo38 (melanoma), SK-ChA-1 (cholangiocarcinoma), and Mz-ChA-1 (cholangiocarcinoma). Furthermore, the additional derivatives **22**, **24**, and **128,** which have not yet been studied elsewhere, were tested. Cells were grown in the presence or absence of increasing concentrations of each derivative, and the IC50 concentration was determined after 72 h (Table 1).

CUR and its derivatives had a cytotoxic effect on the panel of tumor cell lines tested and it was possible to obtain the IC50 value from the dose-dependent growth curves. From the comparison of the IC50, all derivatives were more active than CUR on the CML-derived K562 cell line, while on the other tumor cell lines tested, they showed cytotoxicity that was comparable, slightly higher, or even worse than CUR. Therefore, the modifications introduced seemed to give the derivative selectivity towards the K562 cell line. On these cells, derivatives **2** and **22** had identical cytotoxicity (IC50 = 0.5 ± 0.1 µM), about 34 times more than CUR (IC50 = 17 ± 1 µM). The derivatives **40**, **41**, **42**, and **128** had slightly higher IC50 values, between 1.2 and 1.9 times more than CUR. The derivative **2** was more active with respect to CUR also in the TT and MG63 cell lines by about 3-fold. Similar behaviour was previously seen in HA22T/VGH and MCF7 cancer cell lines but with higher IC50 values (CUR vs derivative **2**: 17.4 ± 1.2 vs. 12.8 ± 1.5 on HA22T/VGH; 29.3 ± 1.7 vs. 13.1 ± 1.6 on MCF7) [23]. In contrast, derivative **22** had cytotoxicity comparable to CUR in the TT and MG63 cell lines. Therefore, while derivative **2** showed a broad spectrum of action, the strong cytotoxic effect of derivative **22** was restricted to the CML-derived K562 cell line.

With regards to the structure–function relationship, the cytotoxicity of the derivatives on the K562 cell line improved by replacing the bis-ketone with an isoxazole (in **2** and **22**, but not in **24**), an β-enamine ketone (in **40**, **41**, and **42**) or an oxime (in **128**). Among the isoxazole derivatives, the 3-methoxy group in the aromatic rings did not affect the cytotoxicity in CML cells, since its removal in derivative **22** gave rise to a molecule as potent as derivative **2**. Conversely, the presence of the 4-hydroxyl group could be determinant. In fact, in derivative **24**, which lacks this group, the modifications introduced made the molecule very stable, and probably resistant to glucuronidation, but worsened the cytotoxicity. Therefore, the presence of a 4-hydroxyl group has to be considered in designing future isoxazole derivatives. As for derivatives containing a β-enamine ketone (**40**, **41**, and **42**) or oxime (**128**), they were always less active than isoxazole derivatives on CML-derived cells.

Taken together, these results indicated that the molecules with the greatest cytotoxicity in CML cells were derivatives **2** and **22**, with the latter being particularly cytotoxic to CML cells. Interestingly, in clinical studies, CUR has reached blood levels between 0.4 and 3.6 μM [15]. Therefore, if derivatives **2** and **22** were able to reach levels of bioavailability even only comparable to that of CUR, they should also reach the cytotoxic concentration in vivo.

### 2.3. Analysis of Ddistinctive Chemical–Physical Parameters of CUR Derivatives

The comparison of distinctive chemical–physical parameters of a potential drug with those of drugs already on the market makes it possible to predict its bioavailability in silico. For molecules whose parameter values do not exceed the limit, a good bioavailability can reasonably predicted [24,25,26]. The values obtained for the CUR and its derivatives are reported in Table 2.

The results show that CUR, which has a known limited bioavailability, exceeded the number of rotating bonds. As for its derivatives, some of them showed better or worse chemical–physical characteristics. In derivative **42**, the logP and the number of rotating bonds were over the limit and therefore the molecule had worse chemical–physical characteristics than CUR. As for CUR and derivatives **24, 40, 41, 42, 128**, the molecules exceeded the number of rotating bonds; therefore, these derivatives did not represent an improvement in terms of bioavailability. Notably, derivatives **2** and **22** had better chemical–physical characteristics since these complied with all the parameter limits.

### 2.4. Cell Cycle Effects of Physiological Concentrations of CUR and Its Derivatives

**Figure 3:**
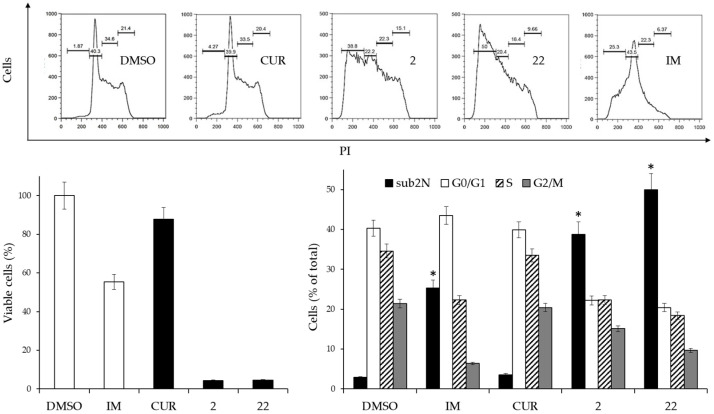
Effects of 2 µM CUR and derivatives **2** and **22**, or 0.15 µM IM on the cell cycle of K562 cells. (**Left side**, **lower panel**): Cell viability after 3 days of treatment. Viable cells are expressed as the percentage of viable cells following treatment compared with viable cells in the DMSO-treated control. Reported values are arithmetic mean ± SD. White columns: negative (DMSO) and positive (IM) controls. (**Upper panel**): Scatterplots of a representative experiment obtained by flow cytometry. (**Right side, lower panel**): Analysis of cell cycle distribution of two experiments performed in triplicate. The values are expressed as arithmetic mean ± SD. sub2N, sub-diploid peak. Asterisks indicate significant values of the sub2N population with respect to DMSO-treated cells (*p* < 0.05).

The effects on the cell cycle produced by a physiological concentration of CUR and its derivatives were compared. To this end, K562 cells were cultured for 72 h in the presence of 2 µM CUR or derivatives **2** and **22**. This concentration was chosen as it represented an intermediate blood value of CUR found in clinical studies. As a reference, 0.15 µM IM (IC50 value) or DMSO was used. The cells were divided into two aliquots. The first was stained with PI and the cell cycle distribution was analyzed by flow cytometry (Figure 3).

The results indicated that derivatives **2** and **22**, at a 2 µM concentration, had marked cytotoxicity associated with a catastrophic effect on the cell cycle. A very pronounced increase in the sub-diploid peak indicated cycle exit and cell death, as occurred in cells that had undergone IM treatment. In contrast, CUR at this concentration did not produce significant differences compared to control cells treated with DMSO alone.

**Figure 4:**
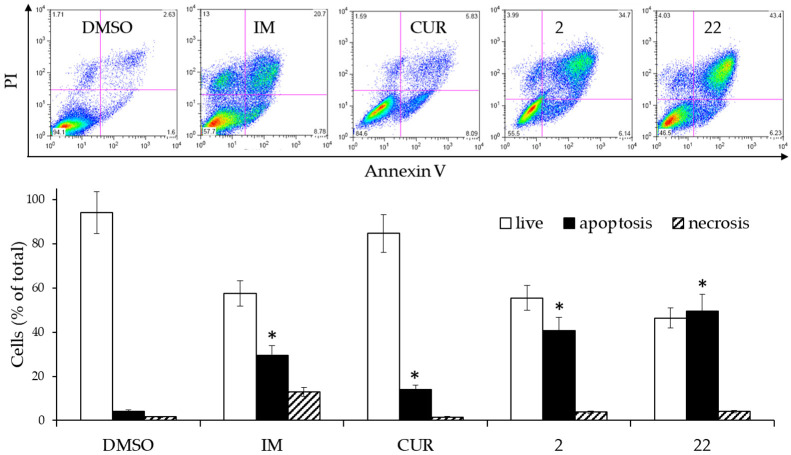
Effects of 2 µM CUR and derivatives 2 and 22, or 0.15 µM IM on the cell death of K562 cells. The cells were treated for 3 days and then analyzed by flow cytometry after staining with Annexin V/PI. (**Upper panel**): Scatterplots of a representative experiment. (**Lower panel**): Analysis of cell death from two experiments performed in triplicate. The values are expressed as arithmetic mean ± SD. Asterisks indicate significant values of apoptotic population with respect to DMSO-treated cells (*p* < 0.05).

Furthermore, it was evaluated whether the derivatives were able to trigger apoptosis. For this purpose, the cells from the second aliquot were stained with AnnexinV and processed by flow cytometry (Figure 4)

The derivatives **2** and **22** triggered significantly elevated levels of apoptosis (sum of early and late apoptosis) compared with cells treated with DMSO alone as well as the control sample treated with IM. Treatment with CUR also showed a significant difference in apoptotic cells, mainly due to the increase in the number of still viable cells in the early stage of apoptosis. For derivatives **2** and **22**, the level of necrotic cells was comparable to that present in the DMSO-treated control sample. From a speculative point of view, these results suggested that if the derivatives were to have bioavailability values even comparable to the CUR, they should exert a more important pharmacological effect than the latter.

### 2.5. Reversal of Drug Resistance in an Imatinib-Resistant K562 Cell Line

**Figure 5:**
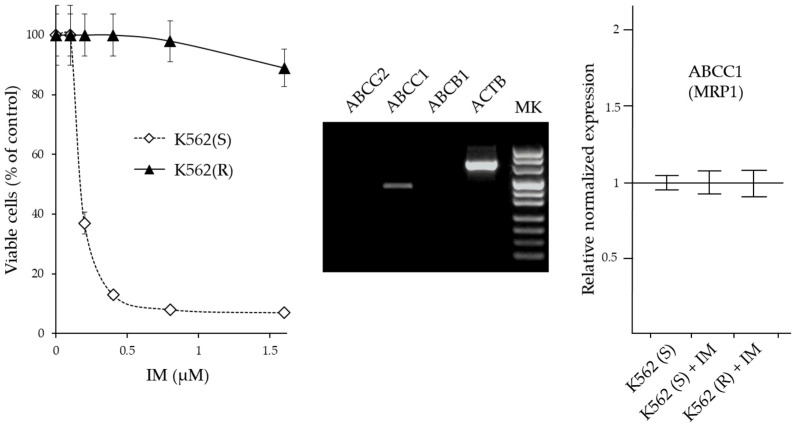
Characterization of drug resistance in IM-resistant K562 cells. **(Left panel):** Cytotoxicity of IM on IM-sensitive and IM-resistant K562 cells. The cells were treated for 72 h with increasing amounts of IM and the number of viable cells was expressed as a percentage of the value observed in the absence of treatment. The values are expressed as arithmetic mean ± SD. (**Middle panel)**: Agarose gel analysis of end-point RT-PCR products of ABC transporter genes (*ABCG2*, *ABCC1*, and *ABCB1*) obtained by 30 cycles of amplification of K652(R) cDNA. *ACTB* = β-actin gene. (**Right panel)**: RT-qPCR analysis of the expression of genes coding for MRP1. The results were expressed as arithmetic mean ± SD (N=6). IM = imatinib.

We next evaluated whether derivative **22**, showing the highest specificity for CML cells, could reverse drug resistance in IM-resistant CML cells. To this aim, we first selected a population of IM-resistant (R) K562 cells by selection in medium containing progressively increasing amounts of IM, as previously described [27]. Firstly, a cell viability assay (MTT) highlighted the drug resistance in K562(R). The K562(R) and K562(S) cells were grown for three days in the presence of increasing concentrations of IM (Figure 5).

Cell viability of K562(S) was already drastically reduced in the presence of 0.5 µM IM, while at this concentration K562(R) cells were still viable and remained viable in the presence of IM concentrations higher than 1.5 µM. In subsequent experiments, 0.5 µM IM was always added to the medium of K562(R) cells.

**Figure 6:**
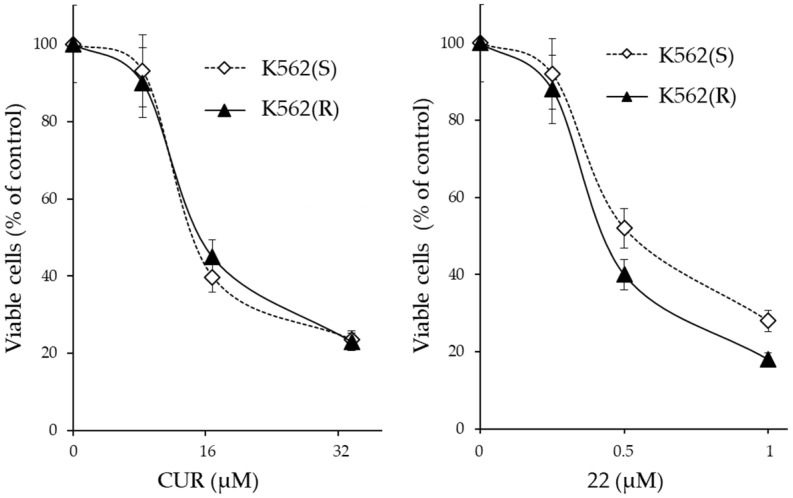
Cytotoxicity of CUR and derivative **22** on IM-sensitive and IM-resistant K562 cells. The cells were treated for 72 h with increasing amounts of the indicated molecule and the number of viable cells was expressed as a percentage of the value observed in the absence of treatment. The values are expressed as arithmetic mean ± SD.

To verify whether the acquired resistance of K562(R) was due to the increased expulsion of the drug, the expression of the genes *ABCB1* (MDR1), *ABCC1* (MRP1) and *ABCG2* (BCRP) which are commonly implicated in drug resistance was evaluated by RT-PCR. After 30 cycles of amplification, the products were visualized by agarose gel electrophoresis. Only *ABCC1* was appreciably expressed in these cells. Subsequently, by Real Time RT-qPCR analysis, the expression levels of this gene were compared in K562(S) before and after treatment with 0.5 µM IM and in K562(R) cultured in the presence of 0.5 µM IM. The expression level of *ABCC1* in K562(S) cells was comparable to that present in K562(R) cells and did not undergo changes in levels following IM treatment. Therefore, the IM-resistance acquired by K562(R) cells did not appear to be associated with increased drug excretion, but rather was attributable to other mechanisms. The cytotoxicity of CUR and its derivative **22** on K562(R) and K562(S) cells was studied in a way similar to that described for IM (Figure 6).

From the results, it emerged that CUR and derivative **22** were cytotoxic in both IM-sensitive and IM-resistant cells. Although the cytotoxic concentrations of CUR and derivative **22** were different at >32 µM and >1 µM, respectively, each of the two molecules was equally active in both IM-sensitive and IM-resistant cell lines. Therefore, both CUR and derivative **22** were capable of reversing IM-resistance in CML cells. However, CUR was effective only if administered in high concentrations (>32 µM), which greatly exceeded the maximum concentration observed in clinical trial patients for this molecule (3.6 µM); meanwhile, derivative **22** was already effective at 1 µM, a value easily achievable in vivo if one also considers its expected better chemical–physical characteristics compared to CUR.

**Figure 7:**
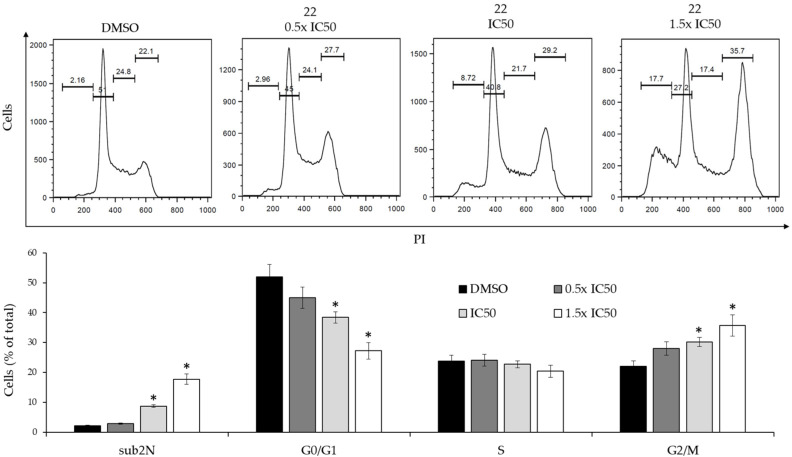
Dose-dependent effects of derivative **22** on the cell cycle of K562(R) cells. (**Upper panel)**: Flow cytometry profiles of a representative experiment obtained by PI staining. (**Lower panel)**: Analysis of cell cycle distribution of two experiments performed in triplicate. The values are expressed as arithmetic mean ± SD. sub2N, sub-diploid peak. Asterisks indicate significant values with respect to DMSO-treated cells (*p* < 0.05).

The dose-dependent effects of derivative **22** on the cell cycle in imatinib-resistant cells were then studied. The K562(R) cells were cultured in the presence of increasing drug concentrations of derivative **22**, ranging from 0.5 times to 1.5 times its IC50 value (Figure 7).

**Figure 8:**
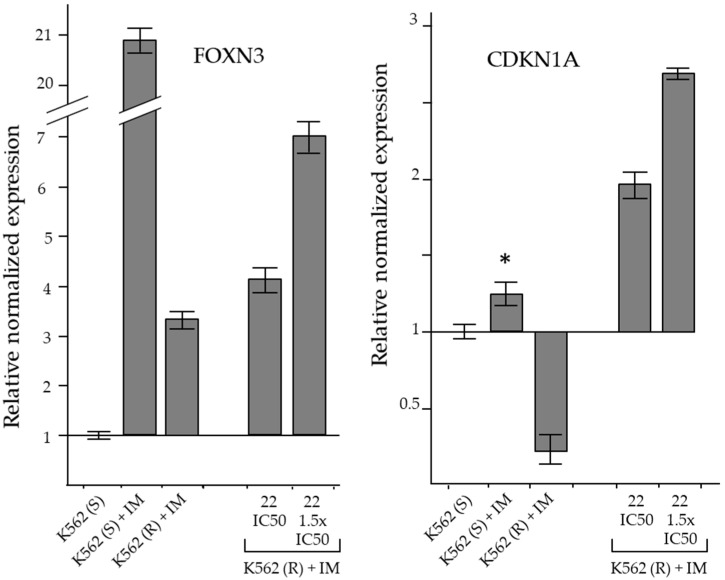
RT-qPCR analysis of the expression of genes coding for cell cycle inhibitory proteins. The results were expressed as arithmetic mean ± SD (n = 6). IM = imatinib. Asterisk = significant value with respect to DMSO-treated K562(S) cells (*p* < 0.05).

The derivative produced significant cell cycle changes, characterized by reduction of the G0/G1 peak, increase of the G2/M peak and subdiploid peak, which were consistent with a G2/M-phase cell cycle arrest. In addition, the modifications produced by derivative **22** on the expression level of the *CDKN1A* and *FOXN3* genes, which code for important cell cycle regulators, were studied by real time RT-qPCR (Figure 8).

The *CDKN1A* gene encodes for p21 (CIP1/WAF1), a protein capable of inhibiting all cyclin/CDK complexes and regulating G1/S and G2/M checkpoints [28,29,30]. Dysregulated cyclin-dependent kinase inhibitor 1A (*CDKN1A*) gene expression was associated with drug resistance [31,32,33]. *FOXN3* (forkhead box N3; check point suppressor 1) belongs to the forkhead box (FOX) protein family. *FOXN3* is involved in cell cycle regulation and its expression decreased in many types of cancers [34]. *FOXN3* expression facilitated DNA damage repair and G2/M-phase arrest [35].

In K562(S) cells, the treatment produced a small but significant increase in CDKN1A expression, whereas in IM-resistant cells its level decreased even in the presence of IM. The reduction of *CDKN1A* expression in IM-resistant CML cells was compatible with a drug-resistance mechanism supporting cell proliferation. Therefore, the reversing effect of derivative **22** on IM-resistance that determined the accumulation of cells in the G2/M phase was consistently associated with a significant increase in *CDKN1A* expression (Figure 8).

In K562(S), IM in cytotoxic concentrations produced a marked increase in *FOXN3* expression, associated with the reduction of cell proliferation. Furthermore, in K562(R), which were insensitive to the same cytotoxic concentration of IM at which they normally proliferated, the increase in *FOXN3* level was quite small and associated with the resumption of proliferation. In resistant cells, cytotoxic concentrations of derivative **22** increased *FOXN3* expression in association with G2/M-phase cell cycle blockade (Figure 8).

Therefore, the cytotoxic and apoptosis-inducing effects of derivative **22** could be due to the increased transcription of the *FOXN3* and *CDKN1A* genes, through a yet unclear mechanism.

## 3. Discussion

Curcumin (CUR) is a natural molecule suggested for the treatment of many human pathologies [3]. Many studies have shown that it has anti-proliferative and anti-carcinogenic activities in animal and human cell lines [4,5]. However, CUR does not reach sufficient blood levels to have an effective pharmacological effect [36]. The inability of the molecule to reach effective doses limits its therapeutic application in spite of its multiple molecular targets and enormous anti-tumor potential [6]. From the analysis of clinical trials carried out on solid and blood tumors [36], it emerged that CUR blood concentration in humans oscillates between 0.4–3.6 μM [15], while the biological effects produced in vitro, such as the triggering of apoptosis, require much higher concentrations (5–80 μM) [3]. Our results also confirmed the need for CUR concentrations above 34 µM to obtain an effect on cell proliferation in CML cells.

The modest blood values observed in the clinical trials were due to low intestinal absorption, reduced solubility/stability in biological fluids, and accelerated metabolism. With regards to the latter, CUR is removed from the bloodstream through the glucuronidation of the 4-hydroxyl group of the aromatic ring mediated by UDP-glucuronosyl transferase and the consequent biliary excretion [13,14] but also following its degradation and elimination of by-products through the kidney [12]. In this respect, the derivative **24** containing the substitution of the hydroxyl group with a methyl group in the aromatic rings should lengthen its permanence in the circulation. However, the substitution significantly reduced the cytotoxicity of the derivative compared to CUR, underlining the importance of the 4-hydroxyl group on the intrinsic cytotoxicity of the molecule. In addition, increasing the stability of the CUR could represent a way to increase its blood level. In fact, at slight alkaline physiological pH, CUR was unstable due to the presence of the bis-ketone group [7,20,37,38]. Therefore, we explored the improvements obtained after replacing the bis-ketone moiety with isoxazole, β-enamine ketone, and oxime. All substitutions stabilized the molecule in physiological pH albumin solution and, at the same time, improved the cytotoxicity of the derivatives on the K562 cell line. These features suggested their possible improved stability and cytotoxicity in biological fluids such as plasma. In particular, for the isoxazole derivatives, which were all stable, the presence of the 4-hydroxyl group, but not of the 3-methoxy group, was decisive for the cytotoxicity of the molecule in CML cells. The derivatives **2** and **22** were particularly cytotoxic to CML cells with IC50 values of 0.5 µM, which suggest they could possibly also reach cytotoxic concentrations in vivo. From a speculative point of view, if these derivatives had a bioavailability even comparable to CUR, which in clinical studies reached blood levels between 0.4 and 3.6 μM [15], the 2 μM concentration at which derivatives **2** and **22** exerted their cytotoxic, cell cycle inhibitory, and pro-apoptotic effects in vitro, could also be easily achieved in vivo. Furthermore, these derivatives showed better predicted bioavailability than CUR and were more stable, so they could reach even higher blood concentrations.

The therapy for CML improved dramatically with the advent of IM, which is now the first-line therapy drug, inducing long-term survival rates above 90%. However, about a quarter of patients develop resistance at some point during therapy [39]. Drug-resistance phenomena are accompanied by therapy failure even when large doses of the drug are administered [40]. Point mutations in the BCR-ABL kinase domain are the most frequent mechanisms of acquired resistance development [41]. A derivative of CUR, C817, which acts on drug-resistant CML cells, has been recently described. This molecule inhibited the activity of Bcr-Abl and was active in patients who carried mutations in the imatinib binding domain [42]. However, other IM-evasion mechanisms can cause drug resistance.

One of them is the resistance due to the reduced intracellular drug accumulation by increasing its expulsion, as occurs in CML cells overexpressing ATP-binding cassette (ABC) transporters such as P-glycoprotein (*ABCB1*), multidrug resistance protein 1 (*ABCC1*), and the breast cancer resistance protein (*ABCG2*) [43]. Another mechanism of IM resistance is target-independent resistance, in which the survival and proliferation of resistant cells is independent from the Bcr-Abl kinase activity [44]. The complexity of the drug resistance phenomenon requires further studies aimed at identifying new molecules capable of blocking the different evasion mechanisms. CUR is a very interesting molecule for the development of new drugs to be used in IM-resistant patients, since it is not very toxic and is well-tolerated by the human body, and because it has a broad spectrum of action, which allows it to modulate numerous biological pathways. However, as mentioned earlier, it is very unstable and reaches only low levels in the blood. Since derivative **22** was shown to induce apoptosis in IM-responsive CML cells, we evaluated whether it could be also active in IM-resistant CML cells. The derivative **22**, sensitized IM-resistant cells similar to CUR but at sub-micromolar concentrations, showing comparable cytotoxicity in IM-sensitive and IM-resistant cells. This cytotoxicity was due to G2/M-phase cell cycle blockade and activation of apoptosis.

K562(R) cells, selected by us through the progressive culture in increasing IM concentrations, did not show increased expression of the transporter genes *ABCC1*, *ABCB1* and *ABCG2* compared with sensitive cells, so the IM-resistance mechanism was not associated with reduced intracellular drug accumulation by increasing its expulsion. In contrast, IM resistance in K562(R) was associated with dysregulated expression of the *CDKN1A* and *FOX3N* genes, which are implicated in cell survival and cell proliferation [45,46,47]. The *CDKN1A* gene encoded for the p21 (CIP1/WAF1) protein, a cyclin-dependent kinase inhibitor, which is capable of inhibiting all cyclin/CDK complexes and needed for G2/M transition [45]. Deregulated *CDKN1A* expression was associated with cisplatin resistance in non-small cell lung cancer (NSCLC) [31], and with paclitaxel resistance in melanoma [32]. In IM-sensitive K562 cells, the treatment also produced a small significant increase in *CDKN1A* expression, whereas in IM-resistant cells its level was decreased even in the presence of IM. The reduction of its expression in IM-resistant cells was compatible with a drug-resistance mechanism supporting cell proliferation. In IM-resistant cells, treatment with derivative **22** significantly increased *CDKN1A* expression, reversing the suppression that occurs during resistance acquisition. In a similar manner, in NSCLC, cisplatin-pemetrexed combined treatment upregulated *CDKN1A* expression [33]. Since the treatment with **22** was associated with G2/M arrest and triggering of apoptotis, we believe it likely that the derivative exerted its cytotoxic effect through upregulation of *CDKN1A*. The *FOXN3* gene belongs to the forkhead box (FOX) protein family. *FOXN3* is involved in cell cycle regulation and facilitates G2/M-phase arrest [35]. Its expression is reduced in many types of cancers and its upregulation decreased protein synthesis and cell proliferation in tumor cell lines, while reduced levels increased tumor cell proliferation [34,47]. Accordingly, in K562(S), IM cytotoxic concentrations produced a marked increase in FOXN3 expression which was associated with reduced cell proliferation. In K562(R), which were insensitive to the same cytotoxic concentration of IM, the increase in *FOXN3* level was quite small and associated with the recovery of proliferation. Therefore, the downregulation of this gene was part of the mechanism leading to IM resistance. In resistant cells, the treatment with derivative **22** upregulated *FOXN3* expression, an event associated with G2/M-phase cell cycle accumulation, thus reverting the IM resistance.

## 4. Materials and Methods

### 4.1. Cell Lines and Culture Conditions

The CML-derived K562 cell line was isolated in 1970 from a 53-year-old patient affected by chronic myeloid leukemia in the terminal blast phase [48]. The cell lines SK-ChA-1 and MZ-ChA-1 were originally isolated from undifferentiated and well-differentiated primary cholangiocarcinoma of the extrahepatic bile ducts, respectively. [49] The TT cell line was established from a 77-year-old patient with medullary thyroid cancer [50]. Osteosarcoma cell lines were isolated from a 14-year-old patient (MG63) and a 13-year-old patient (TE85) [51]. The Colo38 cell line was isolated from a 67-year-old female patient affected by melanoma [52]. The K562 cell line was grown in RPMI, while the other cell lines were grown in DMEM. Cell culture media were completed with 10% (*v/v*) fetal bovine serum (FBS), streptomycin 100 μg/mL, and penicillin 100 U/mL. Cultures were maintained in logarithmic phase at 37 °C in a humidified atmosphere containing 5% CO_2_.

### 4.2. Cytotoxicity Assays

The K562 cells were collected by 5 min centrifugation at 1000*× g* and suspended in fresh complete medium. For the other cell lines, the cells were detached by trypsin and then seeded in fresh complete medium, and after 8 h, the medium was replaced with fresh medium. DMSO or serial dilutions of the examined molecule were added to the medium and after 72 h, 100 μL of 0.5 mg/mL 3-(4,5-dimethylthiazol-2-yl)-2,5-diphenyl tetrazolium bromide (MTT) solution was added to the culture medium and the cells were incubated for 4 h at 37 °C. Then, 100 μL DMSO were added to each well to dissolve the formazan crystals. Viable cells were quantified by measuring photometric absorbance at 570 nm using a multi-well plate reader. Alternatively, the optical count of cells in Burker’s chamber was performed. The IC50 value was estimated by using the Excel add-in ED50V10. Three independent experiments were performed in triplicate for each condition.

### 4.3. Cell Cycle Analysis

The cell cycle was investigated after a 72-h treatment with the test substances. The cells, collected by centrifugation or by trypsin, were fixed in cold 70% ethanol for 20 min. After washing in PBS, they were suspended in 0.2 mL PBS containing RNase A and propidium iodide (PI) for 30 min at 37 °C in the dark. The percentage of cells in the different phases of the cell cycle was determined by a BD FACSCalibur flow cytometer (BD Biosciences, San Josè, CA, USA). The cell cycle analysis was performed using the FlowJo software, version 9.9.6 (Tree Star, Ashland, OR, USA). The experiments were repeated at least two times in triplicate.

### 4.4. Determination of Apoptotic Cell Death

Apoptosis was determined using flow cytometry after Annexin V and Propidium Iodide (PI) dual staining. Cultured cells were incubated in 6-well plates in medium containing the examined molecule for 72 hrs. After this time, the control cells growing in a normal medium reached 70–80% of confluence. The harvested cells were stained using an Annexin V/PI Kit (MabTag, Friesoythe, Germany) according to the manufacturer’s protocol, and immediately analyzed by flow cytometry as previously described [53]. All samples were assayed in triplicate and each experiment was performed at least two times.

### 4.5. UV−Visible Spectrum Analysis

Absorbance readings were taken from 220 to 600 nm using a Perkin-Elmer spectrophotometer (Waltham, Massachusetts, USA). In the experiments of UV−visible absorption, CUR and its derivatives were diluted to 25 μM in 0.1 M phosphate buffer pH 7.2, in the presence or absence of 2 µM albumin from bovine serum. The spectra were collected at 5 min intervals in the first 20 min of incubation and then at 40, 80 and 160 min.

### 4.6. Real Time Quantitative PCR Analysis

RNA isolation was carried out by using guanidine isothiocyanate (TRIzol reagent, Invitrogen Corporation, Carlsbad, CA, USA). Cells were lysed in 1 mL of TRIzol reagent and then 200 µL of chloroform was added. The mixture was vigorously shaken, incubated at room temperature for 10–15 min and centrifuged at 12,000× g for 15 min. The aqueous phase was collected, and the RNA was precipitated by isopropanol addition. The pellet, previously washed in 75% ethanol, was dissolved in 10 mM Tris-HCl pH 7.5, 1 mM EDTA. Purified RNA samples were processed with the DNA-free DNA Removal Kit (Invitrogen, Thermo Fisher Scientific, Milan, Italy) to remove contaminating DNA. RNAs (2 µg) were reverse transcribed (RT) with or without the ImProm-II™ Reverse Transcriptase (Promega Italia, Milan, Italy) using oligo-dT primers in a standard 20 µL reaction. Then, 1 µL of this reaction mixture was used as a template in gene-specific amplifications performed on a StepOnePlus Real-Time PCR System using the StepOne software v2.3 (Thermo Fisher Scientific, Milan, Italy). The sequence of primers used in the amplification of *FOXN3*, *CDKN1A* and *ACTB* mRNAs have been previously described [27]. The sequence of primers used in the amplification of *ABCB1*, *ABCC1*, and *ABCG2* mRNAs have been previously described [54]. PCR amplifications were performed in a 50 µL volume containing 25 µL SYBR green PCR master mix (Thermo Fisher Scientific, Milan, Italy) containing the ROX internal passive reference dye, 0.5 µM of each primer, and optimized MgCl_2_ concentration between 1.5 and 3 mM. All determinations were performed in triplicate wells. Samples in which the RNA was not reverse-transcribed gave CT values comparable to those obtained in the no-template control well. Endpoint amplified products were subjected to melt-curve analysis. The relative quantity of the target transcript in the sample was calculated with respect to the reference ACTB mRNA using a comparative CT (ΔΔCT) method. The relative value was expressed as 2^−ΔΔCT^.

### 4.7. Selection of IM-Resistant K562 Cells

The IM-resistant cells were isolated as described previously [27]. The K562 cells initially sensitive to the drug, herein cited as K562(S), were cultured in complete culture medium in the presence of 0.1 µM IM for 15 days. They were then diluted in fresh medium and grown in the presence of increasing IM concentrations, doubling it every 15 days. After 4 months, the resistant K562 cells, indicated as K562(R), were capable of proliferating in the presence of 2 µM IM. To maintain the resistance characteristics, the cells were cultured in the constant presence of 0.5 µM IM.

### 4.8. Bioavailability Prediction Analysis

Parameters such as molecular weight (≤500), presence of hydrogen-bond donor groups (≤5), presence of hydrogen-bond acceptor groups (≤10), octanol/water partition coefficient (Log P, ≤5), were calculated as part of Lipinski’s five rule [24]. In addition, polar surface area (PSA, acceptable values ≤ 140 Å) [25] and the molecular flexibility, measured based on the number of free rotatable bonds present in the structure (acceptable values ≤7) [26], were considered. All the parameters were calculated by using the Molinspiration Cheminformatics Software version 2014.11.

### 4.9. Statistical Analysis

The results were expressed as the arithmetic mean ± standard deviation. Statistical calculations were performed using a one-way ANOVA, and the differences among groups were examined using the Bonferroni *t*-test. The *p*-values < 0.05 were considered significant.

## 5. Conclusions

The CUR isoxazole derivative **22** has chemical–physical and biological characteristics suitable for reaching pharmacological blood levels in human plasma, and is also effective in blocking the proliferation and triggering apoptosis of CML cells in vivo. Furthermore, it is capable of sensitizing IM-resistant CML cells. Therefore derivative **22** could represent a potential drug for the experimental therapy of CML and drug-resistant forms, alone or administered in combination with IM.

## Figures and Tables

**Table 1:** Cytotoxicity of CUR and its derivatives on cancer cell lines. Viable cells were evaluated 72 h after treatment with increasing concentrations of the indicated molecules. The values indicate the IC50 concentration (μM) ± SD. Values in red indicate a greater cytotoxicity than CUR.

	K562	Colo38	TT	MG63	TE85	SK-ChA-1	Mz-ChA-1
**CUR**	17.1 ± 1.0	13.6 ± 0.7	13.5 ± 0.9	8.7 ± 0.7	9.5 ± 0.7	4.6 ± 0.3	7.6 ± 0.6
**2**	0.5 ± 0.1	13.7 ± 0.8	4.9 ± 0.2	3.0 ± 0.2	16.4 ± 0.9	4.1 ± 0.3	10.4 ± 0.6
**22**	0.5 ± 0.1	16.1 ± 1.0	15.7 ± 1.1	18.0 ± 1.0	11.8 ± 0.7	36.1 ± 2.1	47.5 ± 2.7
**24**	15.9 ± 0.9	19.3 ± 0.9	8.4 ± 0.5	40.7 ± 2.5	22.9 ± 1.3	14.5 ± 0.9	14.1 ± 0.9
**40**	9.0 ± 0.5	24.1 ± 1.5	27.8 ± 1.8	18.8 ± 1.3	17.9 ± 1.0	13.6 ± 0.7	18.2 ± 1.1
**41**	13.2 ± 0.8	24.4 ± 1.6	23.5 ± 1.5	16.6 ± 0.8	29.3 ± 1.9	16.1 ± 0.8	16.4 ± 0.9
**42**	7.3 ± 0.6	21.5 ± 1.3	23.7 ± 1.3	10.7 ± 0.5	16.8 ± 1.0	10.7 ± 0.6	9.4 ± 0.5
**128**	12.1 ± 0.5	14.9 ± 0.7	19.8 ± 1.3	20.3 ± 1.4	37.7 ± 2.2	3.1 ± 0.2	3.3 ± 0.2

**Table 2:** Molecular physicochemical properties of CUR derivatives. LogP = octanol/water partition; MW = molecular weight; nON = number of hydrogen bond acceptors; nOHNH = number of hydrogen bond donors; nrotb = number of rotatable bonds; PSA = polar surface area. Values that exceeded the limit are indicated in red.

Limit Values		CUR	2	22	24	40	41	42	128
≤5	**LogP**	2.30	4.33	4.70	4.29	4.67	3.95	5.23	3.57
≤500	**MW**	368.38	365.38	305.33	455.51	457.53	409.48	471.55	478.50
≤10	**nON**	6	6	4	8	6	6	6	9
≤5	**nOHNH**	2	2	2	0	3	3	3	2
≤140	**PSA**	93.07	84.96	66.49	77.00	88.02	88.02	88.02	123.62
≤7	**nrotb**	8	6	4	10	10	9	10	11
0	**violations**	1	0	0	1	1	1	2	1

## Data Availability

Not applicable.
